# Dysregulation of nuclear receptor COUP-TFII impairs skeletal muscle development

**DOI:** 10.1038/s41598-017-03475-5

**Published:** 2017-06-09

**Authors:** Hui-Ju Lee, Chung-Yang Kao, Shih-Chieh Lin, Mafei Xu, Xin Xie, Sophia Y. Tsai, Ming-Jer Tsai

**Affiliations:** 1 Department of Molecular and Cellular Biology, Baylor College of Medicine, Houston, TX 77030 USA; 2 Program in Developmental Biology, Baylor College of Medicine, Houston, TX 77030 USA; 3 Department of Medicine, Baylor College of Medicine, Houston, TX 77030 USA; 4 Department of Physiology, College of Medicine, National Cheng Kung University, Tainan, 701 Taiwan, ROC

## Abstract

Chicken ovalbumin upstream promoter-transcription factor II (COUP-TFII) has been shown to inhibit myogenesis and skeletal muscle metabolism *in vitro*. However, its precise role and *in vivo* function in muscle development has yet to be clearly defined. COUP-TFII protein expression level is high in undifferentiated progenitors and gradually declines during differentiation, raising an important question of whether downregulation of COUP-TFII expression is required for proper muscle cell differentiation. In this study, we generated a mouse model ectopically expressing COUP-TFII in myogenic precursors to maintain COUP-TFII activity during myogenesis and found that elevated COUP-TFII activity resulted in inefficient skeletal muscle development. Using *in vitro* cell culture and *in vivo* mouse models, we showed that COUP-TFII hinders myogenic development by repressing myoblast fusion. Mechanistically, the inefficient muscle cell fusion correlates well with the transcriptional repression of *Npnt, Itgb1D and Cav3*, genes important for cell-cell fusion. We further demonstrated that COUP-TFII also reduces the activation of focal adhesion kinase (FAK), an integrin downstream regulator which is essential for fusion process. Collectively, our studies highlight the importance of down-regulation of COUP-TFII signaling to allow for the induction of factors crucial for myoblast fusion.

## Introduction

Skeletal muscle development is a tightly regulated process, including the determination of mesodermal precursors committed into myogenic lineage (myoblasts) and subsequent differentiation and fusion of these cells into multinucleated myotubes. Syncytia myotubes then undergo myofibrillogenesis to become mature myofibers^1, 2^. Myogenic regulatory factors (MRFs) are a family of b-HLH transcription factors responsible for myogenic determination and differentiation. This family of proteins includes four members: *Myf5, MyoD, Myogenin and MRF4/herculin/ Myf6* ^3^. Primary myogenesis is initiated in somites via the spatial and temporal expression of MRFs including *Myf5* and *MyoD*, which are required for initial specification of skeletal myoblasts. *Myogenin* is involved in the differentiation process and is required for myoblast fusion. *MRF4* is expressed transiently in myotomes between days 9 and 11.5 p.c., and functions like *Myf5* and *MyoD* as a myogenic determination gene. *MRF4* reappears in late fetal (day 16 p.c.) and postnatal muscle and in the late stage of *in vitro* myogenesis, suggesting its role in terminal differentiation and maintenance of mature myotubes^3–7^.

Many signaling pathways involved in mammalian myoblast fusion have been identified, including integrin-focal adhesion kinase (FAK), Rho small GTPase, mitogen-activated protein kinases (MAPKs), calcineurin-NFATc2, Wnt, TGF-β and myomaker^8, 9^. Among those signaling molecules, cell adhesion proteins are the major players that regulate cell fusion. Integrins consist of α and β transmembrane subunits, which enable cells to adhere to the extra cellular matrix (ECM) to form the so called focal adhesion^10^. FAK, focal adhesion kinase, could be recruited to clustering integrins (ECM bound) and activates through autophosphorylation at Tyr397^11^. Upon activation, FAK creates a docking site for interaction with other signaling proteins and initiates intracellular signal transduction cascades to promote cell migration, growth and differentiation, thus participating in muscle cell fusion^12–15^. Moreover, FAK activation also enhances Caveolin-3 and integrin-β1D expression, two muscle-specific proteins that play key roles in myoblast fusion^16, 17^.

Chicken ovalbumin upstream promoter-transcription factor II (COUP-TFII), also known as NR2F2, is an orphan member of the nuclear receptor (NR) superfamily. COUP-TFII is widely detected in the mesenchymal compartment of developing organs and is required for appropriate mouse embryogenesis^18–20^. Germ-line inactivation of *COUP-TFII* (*COUP-TFII* ^*−/−*^) is embryonically lethal, whereas *COUP-TFII heterozygous* mice (*COUP-TFII* ^+/−^) are viable and exhibit reduced fat tissue and increased lean muscle mass^21^. Depletion of COUP-TFII in C3H10T1/2 mesenchymal precursor cells led to a preferential lineage switching from adipocyte and chondrocyte to myoblast and osteoblast^22^, suggesting a role of *COUP-TFII* in myogenesis *in vitro* ^23–25^. However, little is known about its *in vivo* function in muscle development and muscular diseases. Recently, we reported that COUP-TFII is a key regulator of satellite cell development and contributes to the etiology of muscular dystrophy^26^. Here, we found that COUP-TFII protein is highly expressed in the myogenic precursors and its expression gradually decreases during muscle cell differentiation, and reaches undetectable levels prior to fusion of myoblasts into mature myotubes. These results prompted us to ask whether downregulation of COUP-TFII expression is required to proceed with muscle development. As such, we generated a mouse model constitutively expressing COUP-TFII protein during myogenesis and found that elevated COUP-TFII activity impaired skeletal muscle development. Using these transgenic mice as well as cell culture systems, we demonstrated mechanistically that COUP-TFII repressed myogenesis through direct transcriptional repression of genes important for myoblast fusion as well as inhibition of FAK activation.

## Results

### Decrease of COUP-TFII expression during myogenesis of C2C12 cells

**Figure 1:**
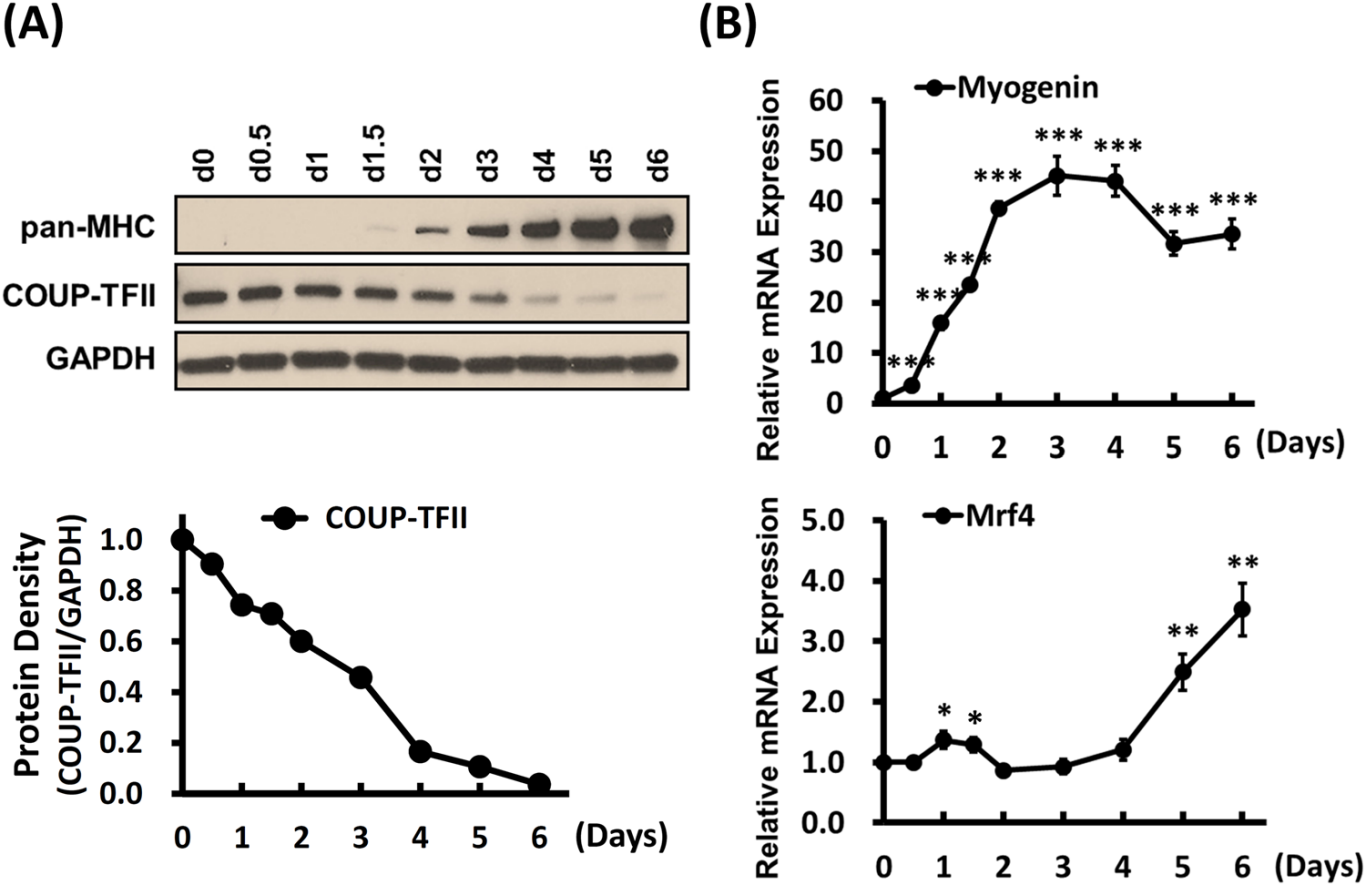
COUP-TFII expression decreases during myogenic differentiation in C2C12 mouse myoblast cells. (**A**) Western blot analyses of COUP-TFII protein expression during myogenic differentiation in C2C12 cells. Relative COUP-TFII protein level was quantified by band density and normalized to GAPDH as shown in bottom panel. (**B**) Quantitative RT-PCR examination of *Myogenin* (MRF in early differentiation stage) and *MRF4* (MRF in terminal differentiated myotube) mRNA expression pattern C2C12 cells were placed in the differentiation medium (DM, 2% horse serum) and incubated for the indicated time points. Day 0, serum withdraws in confluent culture cells as the initiation of myogenesis; medium was changed every other day. Data are shown as mean ± SEM. One-way ANOVA followed by Bonferroni’s Multiple Comparison Test; *p < 0.05, **p < 0.01, ***p < 0.001, compared to day 0.

In order to define the role of COUP-TFII in myogenesis, we first examined the expression of COUP-TFII during muscle cell development in a cell culture system. Proliferating C2C12 myoblasts can be induced to develop into post-mitotic multinucleated myotubes upon serum withdrawal^23^. As shown in Fig. 1A, COUP-TFII protein levels decline during myogenic differentiation of C2C12 cells. Similarly, COUP-TFII mRNA also reduces as early as 12 hr post-differentiation and continuously drops till day 4, the time for cultured myotube maturation (Supplementary Fig. S1). The decreased COUP-TFII expression during myogenesis is tightly correlated with the up regulation of myogenin (Fig. 1B). Myogenin expression increases when the myoblasts start to differentiate into fusion-competent myoblasts and nascent myotubes. The level of myogenin starts to decrease at day 4, while mature myotubes are formed as indicated by the expression of terminal differentiated factor, MRF4. The inverse correlation between the levels of COUP-TFII and myogenic regulators suggest the importance of COUP-TFII down regulation in myogenesis.

### Ectopic COUP-TFII expression inhibited while loss of COUP-TFII accelerated myogenesis in C2C12 cells

**Figure 2:**
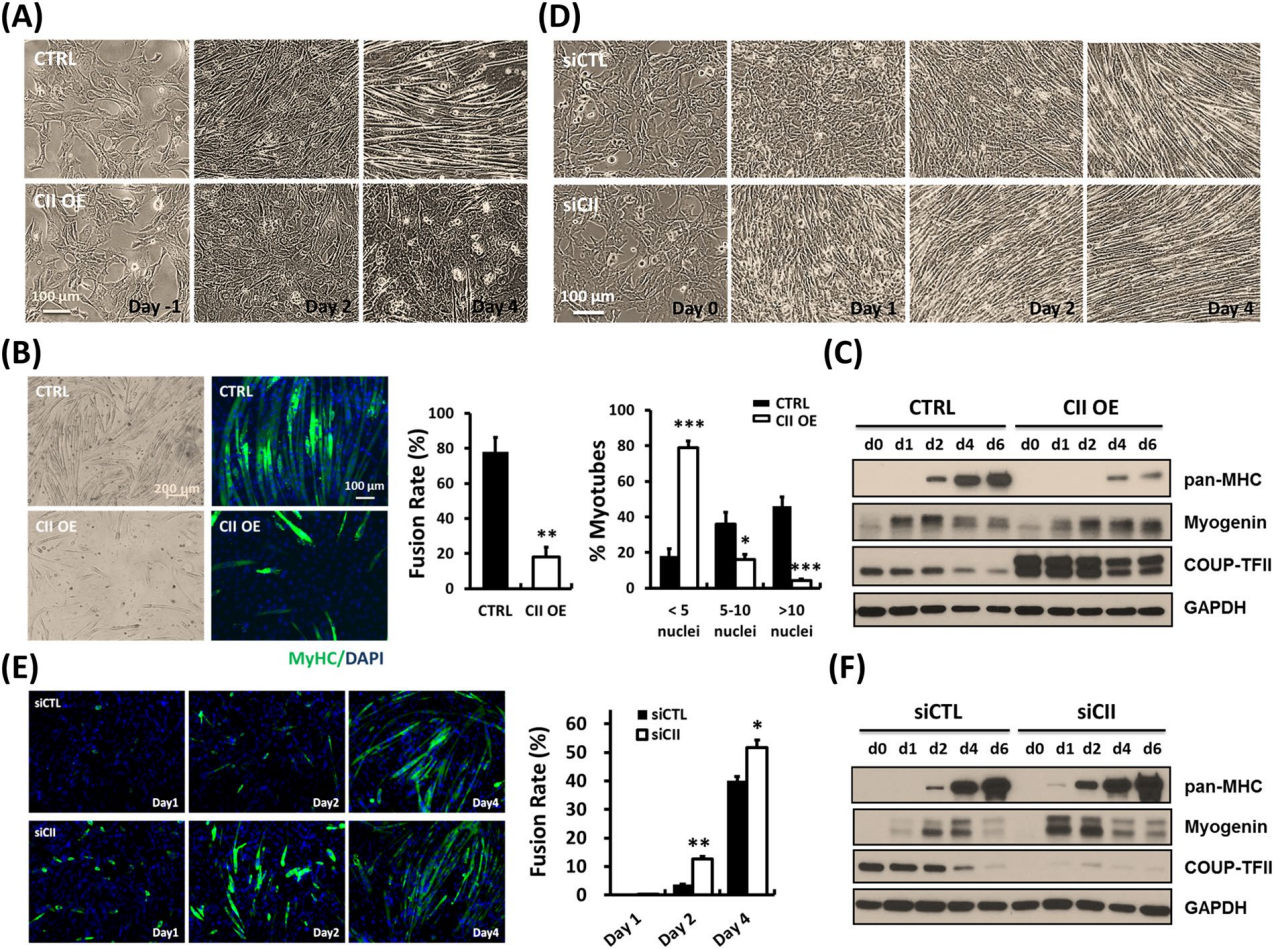
COUP-TFII represses myogenesis in C2C12 mouse myoblast cells. (**A**) Phase contrast images of ectopic COUP-TFII overexpressing and C2C12 cells at the indicated time points during myogenesis. (**B**) Six days post-differentiation, COUP-TFII overexpressing cells were fixed and immunostained for pan-MHC, and then quantified for the fusion rate and the percentage of MyHC+ nuclei present in myotubes with the indicated number of nuclei (Right panels). (**C**) Western blot examination of protein expression patterns in ectopic COUP-TFII overexpressing cells. (**D**) Down regulation of COUP-TFII by siRNA increases differentiation of C2C12 cells as analyzed by bright field images. (**E**) As indicated in post-differentiation days, COUP-TFII silencing cells were stained with pan-MHC antibodies (left panels) and their fusion rates were calculated. (**F**) Protein expression pattern in COUP-TFII silencing C2C12 cells during myogenesis. The cells were harvested with 0.1% SDS-RIPA lysis buffer at the indicated time points under serum withdrawal culture conditions. Equal amount of cell lysates were subjected to immunoblotting with the indicated antibodies. GAPDH served as an internal control. Data are shown as mean ± SEM. Two-tailed Student's *t* test; *p < 0.05, **p < 0.01, ***p < 0.001, compared to control cells.

In order to evaluate COUP-TFII’s role in myogenesis, we over-expressed COUP-TFII in C2C12 cells during myogenesis through retroviral mediated transduction of COUP-TFII expression. As shown in Fig. 2A, phase contrast microscopic imaging clearly indicates that high levels of COUP-TFII repress fusion of myoblasts into myotubes. Mature myotubes or myofibers are multinuclear, which can be observed in FL microscope by immunostaining by pan-MHC (MF20) antibody and counterstained with DAPI for the nuclei. Rare myotube formation indicates defective myogenesis and myoblast fusion in COUP-TFII-expressing C2C12 cells (Fig. 2B left panel). Quantitative analysis indicates that COUP-TFII activation largely impaired myoblast fusion (Fig. 2B middle panel) and those fused fibers contain less nuclei (Fig. 2B right panel). Accordingly, a decreased myosin heavy chain (MyHC) expression indicates that COUP-TFII overexpression prevents C2C12 cells from differentiating into mature myofibers. Consistently, delayed upregulation of myogenin and persisted myogenin expression in late stages also support an impaired differentiation in COUP-TFII overexpressed cells (Fig. 2C). In contrast, silencing of COUP-TFII protein by siRNA 3 days prior to induction accelerates the myogenic process. As presented in Fig. 2D and E, COUP-TFII ablated cells start to differentiate and MyHC expression is detected as early as day 1 post-induction, while control cells have little morphological changes. The difference is even more pronounced at day 2 and myoblast fusion rate also increases upon COUP-TFII ablation. These morphological differences are further substantiated by Western blot analysis, in which COUP-TFII silenced cells shows earlier increase of myogenin and MyHC expression at day 1 and day 2 post-differentiation (Fig. 2F). We also observed that silencing of COUP-TFII can promote myoblast differentiation by repressing cell proliferation (Supplementary Fig. S2). Taken together, these results clearly indicate that down regulation of COUP-TFII during myogenesis is essential for proper differentiation of myogenic cells.

### Myf5-Cre driven COUP-TFII overexpression impairs skeletal muscle development *in vivo*

**Figure 3:**
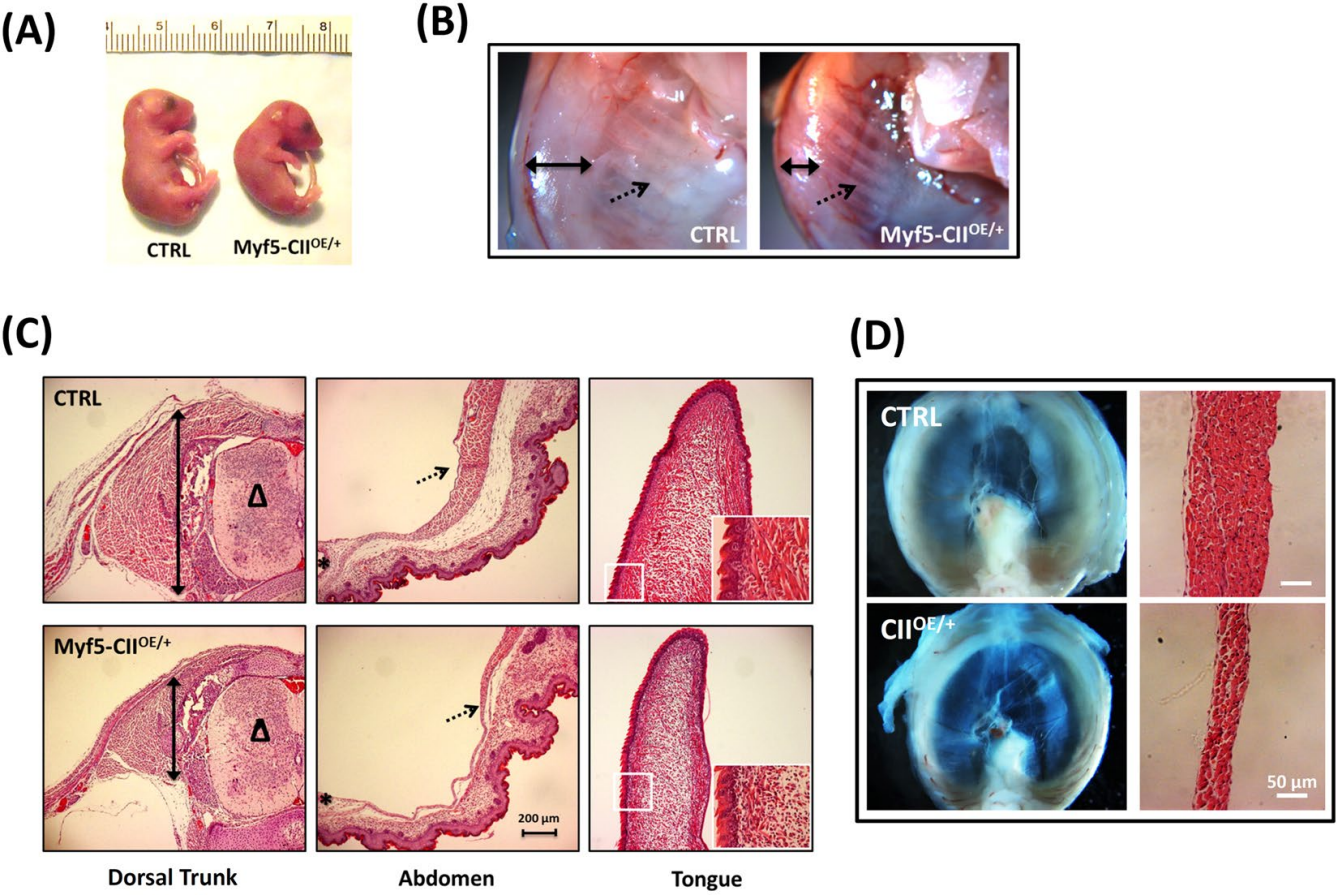
Myf5-Cre driven COUP-TFII overexpression impaired skeletal muscle development *in vivo*. (**A**) Phenotypical differences of control littermate (CTRL) and My5-COUP-TFII^OE/+^ fetus. (**B**) Fixed and de-skinned fetus under dissection microscope. Bold arrow: body wall; dotted arrow: ribs. (**C**) H&E staining of dorsal trunk, abdomen and tongue muscles, sequentially. Bold arrow: the thickness of psoas major muscle; dotted arrow: abdomen muscle; **∆** spinal cord; *umbilical ring. Enlarged square area of the tongue was shown on the bottom right site. (**D**) Dissection microscope imaging of whole diaphragm and H&E staining was shown in right panel. Animals were delivered by cesarean section on the 18^th^ day of gestation.

The aforementioned *in vitro* cell culture results support the inhibitory function of COUP-TFII in myogenesis. We then asked whether COUP-TFII has a similar role *in vivo* using a genetic mouse model to over express COUP-TFII in muscle lineage. For this purpose, we used myogenic progenitor specific Myf5-Cre to remove the “STOP” cassette from LoxP-STOP-LoxP-COUP-TFII transgenic lines to ectopically express COUP-TFII in muscle cells (Myf5-CII^OE/+^) as previously described^27^. We found that all of the Myf5-CII^OE/+^ pups die right after birth. The lethality is due to the inability of new born pups to breathe as indicated by the lack of air in their lungs and the subsequent sinking of the lung to the bottom of a water container (Supplementary Fig. S3). We then dissected the embryo at E18.5 and the Myf5-CII^OE/+^ embryo has a distorted posture with an arched back and weakened limbs (Fig. 3A). After tearing off the paraformaldehyde fixed embryo skin, the Myf5-CII^OE/+^ fetus shows less trunk muscle with visible ribs as compared to its control littermate, indicating the defective development of skeletal muscle in Myf5-CII^OE/+^ mice (Fig. 3B). Histologically, there is much less skeletal muscle in all different regions of Myf5-CII^OE/+^ embryos as compared to control littermates (Fig. 3C, Supplementary Fig. S4). Since Myf5-CII^OE/+^ pups cannot breathe, we analyzed their diaphragm. Indeed, the diaphragm muscles of Myf5-CII^OE/+^ embryos were clearly thinner than that in the control counterpart and H&E staining further confirmed the muscle development defect (Fig. 3D). Therefore, using COUP-TFII transgenic animals, we have demonstrated that ectopic expression of COUP-TFII during myogenesis inhibited skeletal muscle development *in vivo*.

### COUP-TFII overexpression inhibits muscle maturation *in vivo*

**Figure 4:**
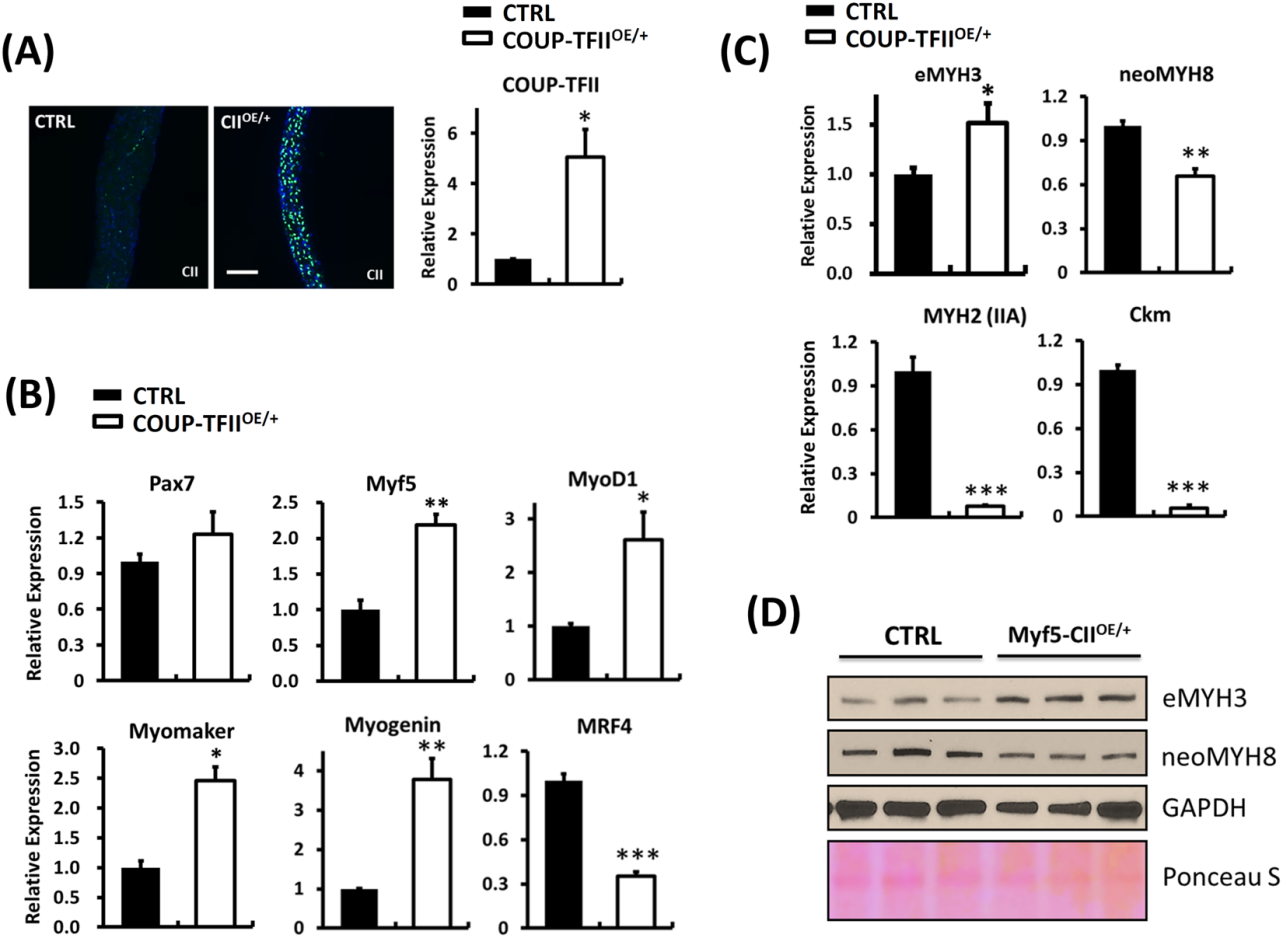
COUP-TFII overexpression inhibits diaphragm skeletal muscle maturation. (**A**) Immunofluorescence staining and quantitative RT-PCR examination of COUP-TFII protein and mRNA expression in the diaphragm. Quantitative RT-PCR examination of (**B**) *Pax7, Myf5, MyoD1, Myomaker*, *Myogenin* and *MRF*4 (**C**) embryonic (e*MYH3*), neonatal (neo*MYH8*) and adult (*MYH2*, IIA) myosin heavy chain, *Ckm* mRNA expression in the diaphragm muscle of control and My5-COUP-TFII^OE/+^ fetus. Total RNA was extracted using TRIzol® reagent. (**D**) Western blot analysis of myosin heavy chain protein expression, eMYH3 and neoMYH8, in the diaphragm muscle of control and My5-COUP-TFII^OE/+^ fetus. Protein was extracted using 0.1% SDS-RIPA buffer. Diaphragm muscle was dissected from E18.5 fetus and tissues were homogenized using a polytron homogenizer. Data are shown as mean ± SEM. Two-tailed Student's *t* test; *p < 0.05, **p < 0.01, ***p <  < 0.001, compared to control mice.

To further characterize the skeletal muscle properties of Myf5-CII^OE/+^ mice, we took the whole diaphragm muscle from E18.5 pups for molecular characterization. COUP-TFII overexpression was confirmed by immunostaining and quantitative RT-PCR (Fig. 4A). Increased *Myf5, MyoD1*, *Myogenin, Myomaker* and decreased *MRF4* expression in Myf5-CII^OE/+^ mice revealed that the diaphragm muscle remained at early differentiation stages and failed to undergo maturation and terminally differentiated into moytubes (Fig. 4B, Supplementary Fig. S5A). Consistently, expression of an early myogenic marker, embryonic myosin heavy chain (*eMYH3*), was increased while the level of maturation markers neonatal (*neoMYH8*), adult myosin (*MYH2*) and muscle form creatinine kinase (*Ckm*) were significantly decreased in Myf5-CII^OE/+^ mice (Fig. 4C). In line with mRNA data, Western blots clearly show higher embryonic myosin and lower neonatal myosin heavy chain protein levels in the Myf5-CII^OE/+^ diaphragm (Fig. 4D). Similarly, *Ckm* protein, a marker of skeletal muscle maturation expression is clearly decreased in COUP-TFII overexpression mice as shown by immunofluorescence staining (Supplementary Fig. S5B). The numbers of muscle fibers are also reduced upon COUP-TFII overexpression (Supplementary Fig. S5C). These results revealed that high levels of COUP-TFII repressed diaphragm muscle maturation, supporting the *in vitro* conclusion that down regulation of COUP-TFII during myogenesis is essential for proper skeletal muscle development.

### COUP-TFII directly regulates myoblast fusion-associated genes: Npnt, integrin β1D and Cav 3

**Figure 5:**
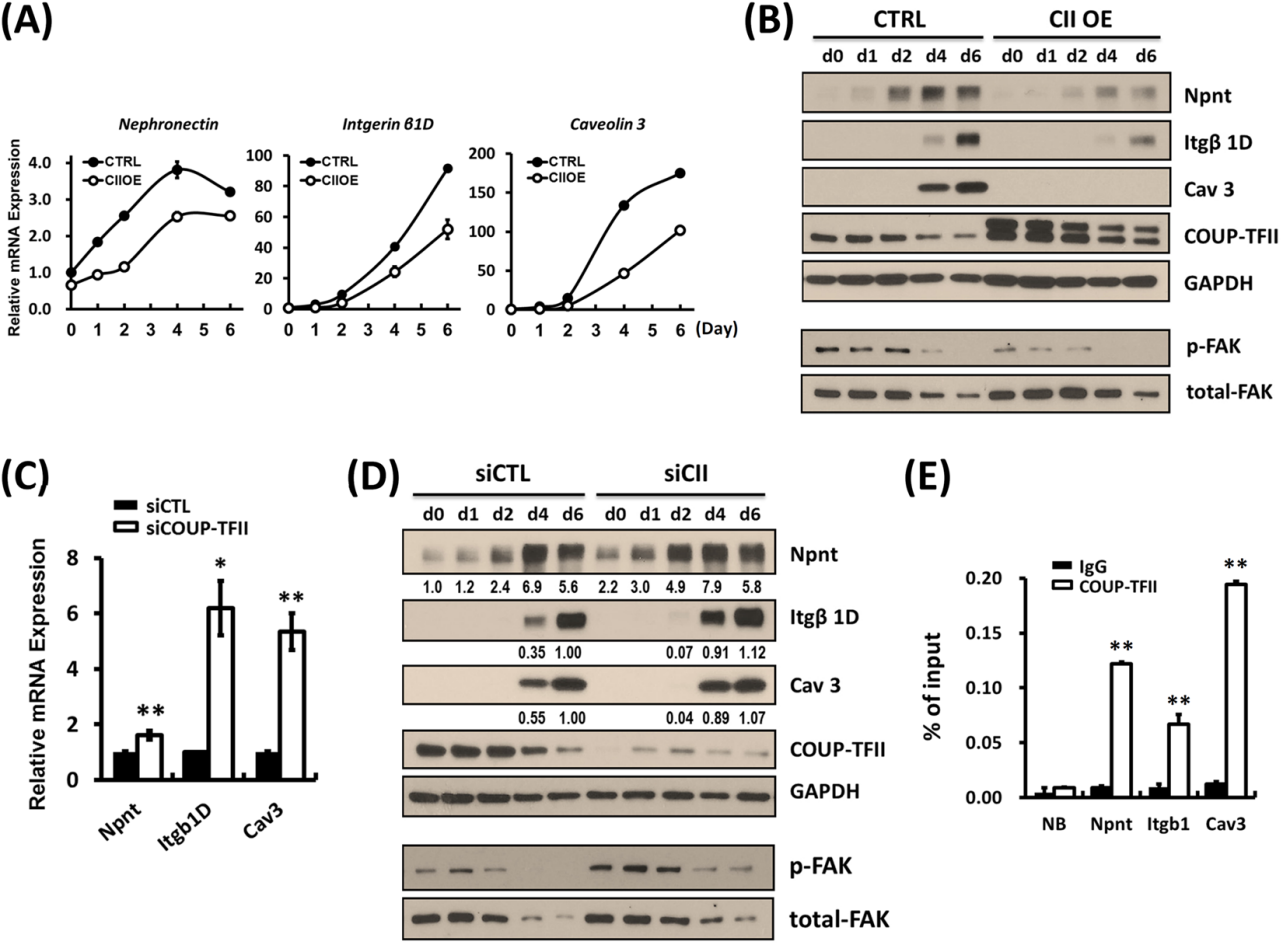
COUP-TFII represses FAK activity and directly regulates expression of moyblast fusion-associated genes in C2C12 cells. Quantitative RT-PCR examination of Nephronectin, Integrin β1D and Caveolin 3 expression in (**A**) Ectopic COUP-TFII overexpressing cells post-serum withdrawal at the indicated time points; and (**C**) COUP-TFII silencing C2C12 cells one day after serum withdrawal. Total RNA was extracted using TRIzol® reagent and mRNA expression levels were quantified by SyBr Green based RT-qPCR analysis. Western blot examination of indicated protein expression patterns in (**B**) ectopic COUP-TFII overexpressing and (**D**) COUP-TFII silencing C2C12 cells during myogenesis. The cells were harvested with 0.1% SDS-RIPA lysis buffer at the indicated time points under serum withdrawal culture condition. Equal amounts of cell lysates were subjected to immunoblotting with the indicated antibodies. Protein expression levels were quantified by densitometry, normalized by GAPDH levels, and shown in the bottom panel as relative values. (**E**) ChIP-qPCR analysis of the recruitment of COUP-TFII to the *Npnt, Itgb1* and *Cav3* enhancer region. Bar graph showed the enrichment of DNA fragments pulled down by COUP-TFII antibody. NB: non-binding region. Data are shown as mean ± SEM. Two-tailed Student’s *t* test; *p < 0.05, **p < 0.01, compared to IgG control.

Next, we want to dissect the underlying mechanism by which COUP-TFII suppresses muscle fiber maturation. In muscular satellite cells, COUP-TFII has been shown to directly repress *caveolin 3* (*Cav 3*), an important molecule for myoblast fusion and a downstream target of integrin-FAK signaling^8, 26^. To define other molecular targets mediating COUP-TFII action for repressing myoblast fusion, we utilized our published ChIP-seq data of COUP-TFII genomic occupancy in mouse atria (GSE46497)^28^, to search for potential COUP-TFII targets involved in myogenesis. We found that COUP-TFII may directly bind to the enhancer/promoter of *Npnt* (Nephronectin, an extracellular matrix protein), *Itgb1* (Integrin β1) and *Cav3* (Caveolin 3) genes. Through RT-qPCR and western blot analyses, we found that COUP-TFII overexpression repressed *Npnt*, *Itgβ1D* (striated muscle specific β1-integrin) and *Cav3* mRNA and protein expression levels (Fig. 5A,B). In the mouse model, COUP-TFII overexpression also downregulated Npnt, Itgβ1D and Cav3 protein expression levels in skeletal muscle (Supplementary Fig. S6). Moreover, silencing COUP-TFII expression activated the transcription of all these genes in proliferating myoblast (Supplementary Fig. S7) and the up-regulation was more significant as early as 1 day post-differentiation (Fig. 5C). Npnt protein expression increases earlier and stays at a high level from day 0 to day 2 as compared to the control. Similarly, Caveolin 3 and integrin β1D protein expression levels were significantly higher in knockdown cells than in control cells 4 days post-serum withdrawal (Fig. 5D). Phosphorylated FAK (pY397), an important regulator of myoblast fusion, was decreased upon COUP-TFII overexpression and was induced in COUP-TFII-deficient cells, suggesting that COUP-TFII negatively suppressed FAK activity to inhibit fusion during myogenesis (Fig. 5B,D). Finally, we showed that COUP-TFII was recruited to the *Npnt, Itgb1* enhancer and *Cav3* proximal promoter in proliferating C2C12 cells using ChIP-qPCR analysis (Fig. 5E). Taken together, COUP-TFII represses myogenesis by directly regulating the transcription of *Npnt*, *Itgβ1D* and *Cav3* genes and suppresses the activation of FAK activity in C2C12 cells.

### Npnt is essential for COUP-TFII silencing mediated acceleration of myogenesis

**Figure 6:**
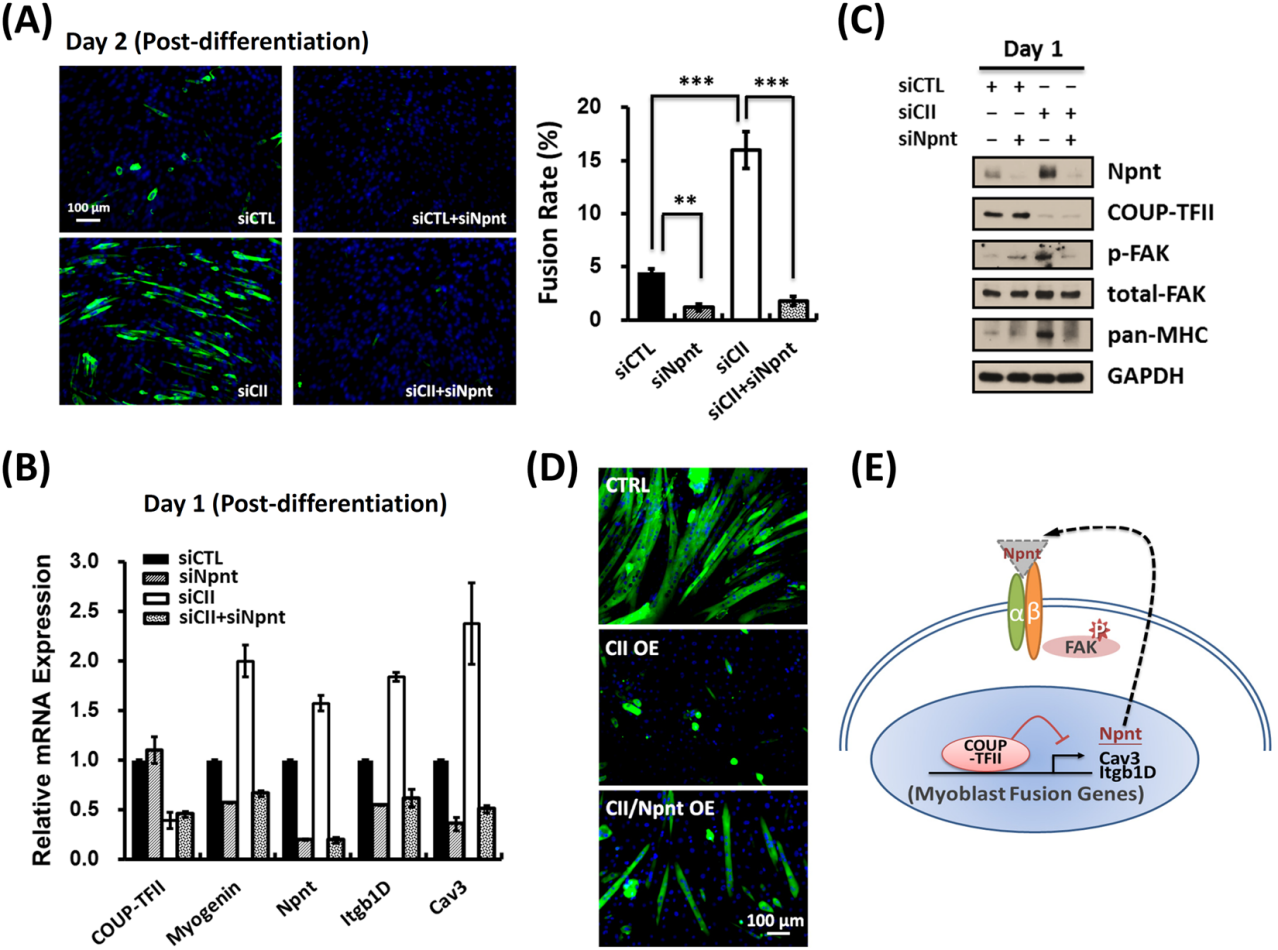
Npnt is essential for COUP-TFII silencing mediated acceleration of myogenesis. (**A**) Two days post-differentiation, indicated cells were fixed and immunostained with pan-MHC (left panel), and their fusion rates were then quantified as the percentage of MyHC+ nuclei versus total nuclei (right panel). (**B**) Quantitative RT-PCR examination of indicated genes expression; (**C**) Immunoblotting of indicated protein expression in control, COUP-TFII silencing, Npnt silencing and double silencing C2C12 cells, one day post-differentiation. Cells were pre-silencing of indicated genes 3 days and reseeded before serum withdrawal. (**D**) COUP-TFII overexpressed cells were infected with control or Npnt overexpressed lentiviruses for three days and the serum was withdrawn for differentiation. Six days post-differentiation, indicated cells were fixed and immunostained with pan-MHC antibodies. (**E**) Illustration of the molecular mechanisms of COUP-TFII-mediated inhibition of myoblast fusion. Data are shown as mean ± SEM. Two-tailed Student’s *t* test; **p < 0.01, ***p < 0.001.

Npnt, an extra-cellular matrix (ECM) protein, can serve as an upstream ligand for activation of the β1-integrin signaling pathway. Upon COUP-TFII ablation, Npnt protein expression level was altered very early, suggesting that Npnt may serve as a key target in mediating COUP-TFII function in myoblast fusion. Therefore, we asked whether Npnt mediates COUP-TFII’s effect in promoting myogenesis. As presented in Fig. 6A, COUP-TFII silencing accelerated myoblast fusion as determined by MyHC staining, and myoblast differentiation was also apparent with cell morphological changes (Supplementary Fig. S8). However, this effect is diminished in the absence of Npnt in C2C12 cells, suggesting that Npnt is essential for COUP-TFII function in regulating myoblast differentiation (Fig. 6A). This conclusion is further supported by RT-qPCR (Fig. 6B) and western analyses (Fig. 6C) in which the effect of COUP-TFII on mRNA expression of *myogenin, Itgβ1D* and *Cav3* and protein expression of MyHC and Cav3 is abolished in the absence of Npnt. Furthermore, as mentioned earlier, activated FAK (p-FAK) was increased in COUP-TFII depleted cells and these effects were lost in double knockdown cells (Fig. 6C). Finally, overexpression of Npnt could partially rescue the fusion deficiency in COUP-TFII overexpressed cells (Fig. 6D). Taken together, we concluded that Npnt is one of the key components responsible for COUP-TFII mediated suppression of myogenesis. Figure 6E illustrates the molecular mechanisms of COUP-TFII-mediated inhibition of myoblast fusion.

## Discussion

The role of *COUP-TFII* in inhibiting myogenesis has been broadly studied in *in vitro* cell culture systems^23–25^; however, little is known about its *in vivo* function. In this study, we show that COUP-TFII is highly expressed in proliferating myoblasts and its level decreases during the differentiation process. We asked whether the reduction in COUP-TFII expression is essential for proper muscle formation. As such, we generated a mouse model ectopically expressing COUP-TFII in myogenic progenitors during myogenesis to investigate the *in vivo* function of COUP-TFII in skeletal muscle development. We also utilized C2C12 mouse myoblast to examine the molecular mechanisms underlying COUP-TFII function in myogenesis.

Myogenesis is initiated in somites via the spatial and temporal expression of *Myf5* and/or *MyoD*, determinant factors for myogenic specification and progression^5^. *Myf-5* transcripts appear as soon as segmentation of somites occurs in 8 days p.c. (post coitum), which is before the expression of all other *MyoD* family RNAs. For this reason, we chose Myf-5 driven Cre recombinase to activate the expression of COUP-TFII at an early stage of myogenesis. As expected, Myf5-Cre driven COUP-TFII activation impaired skeletal muscle development. The skeletal muscle mass is reduced (Fig. 3) and diaphragm muscle cells are not well differentiated as they express embryonic MyHC (*MYH3*) but lack the expression of neonatal MyHC (*MYH8*), adult MyHC (*MYH2*) and *Ckm* in Myf5-COUP-TFII^OE/+^ embryos in comparison to controls (Fig. 4C,D; Supplementary Fig. S2). Surprisingly, COUP-TFII overexpression did not repress MyoD and Myogenin transcription *in vivo*. *MyoD* and *Myogenin* mRNA expressions were significantly higher than littermate controls (Fig. 4B). This result is inconsistent with previous observations in cultured C2C12 cells^23, 24^. This discrepancy is likely due to COUP-TFII inhibition of myoblast fusion, which led to the accumulation of myoblasts that express higher levels of MyoD and Myogenin than the mature myofibers. *Myomaker* gene is expressed in developing skeletal muscle and is subsequently downregulated upon completion of muscle formation^9^. Higher *MyoD1, myogenin, myomaker* and lower *MRF4* expression also indicates that persistent expression of COUP-TFII did not affect progenitor cells differentiating into fusion-competent myoblasts/myocytes, instead it inhibits myoblast and nascent myotube fusion to form mature myotubes^8^. This phenomenon agreed with our previously published results that augmented COUP-TFII activity in satellite cells of developing fibers explicitly blocks cell-cell fusion, resulting in muscle mass reduction in aged mice^26^.

Nephronectin, also called POEM (preosteoblast EGF repeat protein), is an extracellular matrix protein involved in cell-matrix adhesion, which was first reported as a ligand for α8β1-integrin in the kidney^29^ and also serves as a smooth muscle niche in the hair follicle bulge^30^. The abundance of nephronectin increases during myogenic differentiation and it is required for efficient muscle cell fusion *in vitro* ^31^. Our results, for the first time, revealed that COUP-TFII represses Npnt expression through direct binding to the *Npnt* enhancer region (Fig. 5 A,E). Nephronectin has been shown to be important for cardiac tissue engineering^32^, this may also contribute to the heart failure phenotypes exhibited by COUP-TFII over-expression mice^33^. COUP-TFII silencing accelerated myogenic differentiation, as demonstrated by changes of cell morphology into elongated myocytes that express higher levels of genes important for fusion and higher levels of activated FAK (Fig. 5C,D). The upregulation of these molecules in the elongated myoblast indicates that they are competent to fuse into myofibers.

The role of Npnt in mediating COUP-TFII’s effect on myogenesis is quite clear. Npnt is one of the earliest molecules to be upregulated during myogenesis. In addition, in the absence of Npnt, the effect of COUP-TFII in myogenesis is completely abolished, indicating the importance of Npnt in mediating COUP-TFII function (Fig. 6). This result does not exclude roles of other factors, such as Cav 3^34, 35^ and Itgβ1D^36, 37^. They are likely to play important roles as well, since they have been shown to be essential for myoblast fusion^16, 17^. In summary, our study reveals the importance of down regulation of COUP-TFII during myogenesis and that reduction is necessary for the activation of genes, such as *Npnt, Cav3, itgb1D* and activated FAK, to promote myoblast differentiation and fusion into mature myofibers.

## Materials and Methods

### Animals

All experiments involved in animals were approved by Baylor College of Medicine’s Institutional Animal Care and Use Committee (IACUC), under the authority of the Association for Assessment and Accreditation of Laboratory Animals. The mice were housed in the animal facilities of the Center for Comparative Medicine (CCM) of Baylor College of Medicine. All methods were performed in accordance with the relevant guidelines and regulations. The LoxP-stop cassette restrained myc-tagged COUP-TFII expressing (CAG-S-COUP-TFII) mice were generated by our group as previously described^27^. The mice were backcrossed with C57BL/6 mice for at least 5 generations, and maintained according to the National Institutes of Health Guide for the Care and Use of Laboratory. The Myf5-Cre transgenic mice^38^ were purchased from Jackson Laboratory. Myogenic progenitor specific COUP-TFII overexpression mice were generated by crossing CAG-S-COUP-TFII mice with Myf5-Cre mice (Myf5; COUP-TFII^OE/+^).

### Embryo collection, muscle histology and immunohistochemistry (IHC)

E18.5 embryos were collected from the uterus of females. Mouse tissues were dissected, fixed in 4% formaldehyde in PBS for 24 hours, dehydrated and embedded in paraffin. Sections (7 μm) were deparaffinized in xylene, rehydrated and stained with Hematoxylin and Eosin (H&E). Immunostaining was performed with an M.O.M. Kit (Vector Laboratories) following the manufacturer’s protocol. The slides were processed using citrate buffer (pH 6.0) based antigen retrieval and the avidin-biotin peroxidase method. Diaphragm muscle property was analyzed by immunostaining with antibodies to COUP-TFII (R&D), c-myc and Ckm (Santa Cruz Biotech.). Immunofluorescence was conducted using an Alexa Fluor tyramide Kit (TSA; Invitrogen) and sections were counterstained with DAPI (Sigma-Aldrich) and imaging with a Zeiss Axioplan microscope as previously described^39^.

### Cell culture and differentiation

C2C12 mouse myoblast cells (ATCC CRL-1772) were acquired from American Type Culture Collection (ATCC). Cells were cultured in high glucose DMEM supplemented with 110 mg/L sodium pyruvate plus 10% FBS in a 5% CO_2_ humidity incubator. For differentiation, confluent cells were cultured in DMEM medium plus 2% horse serum without sodium pyruvate. Differentiation medium (DM) was changed every other day. All culture reagents were purchased from Invitrogen.

### Expression Plasmids and siRNA transfection

The full-length myc-tagged COUP-TFII cDNA was cloned into pMSCV-puro/neo (Clontech) to generate retroviral based COUP-TFII expression plasmids. Myc-tagged COUP-TFII expression retrovirus were generated and transfected as previously described^40^. Briefly, cells were transduced with retrovirus and cultured with G418 at 1 mg/ml and the selection was stopped as soon as the non-infected control cells died off. SMART pool or single small interfering RNA (siRNA) duplexes targeting COUP-TFII or Npnt and control siRNA were purchased from Dharmacon. Cells were transfected with siRNA duplexes (20–50 nM) using TransFECT 1 siRNA transfection reagent (Dharmacon) according to manufacturer’s instructions.

### RNA isolation and quantitative real-time PCR (RT-PCR)

Total RNA was extracted using TRIzol® reagent according to the manufacturer’s instructions. First strand cDNA was synthesized using 2 μg total RNA and Maxima cDNA synthesis kit (Thermo Scientific). FastStart Universal SYBR Green Master (Rox) reagent (Roche) was used for qPCR and reaction was performed by StepOnePlus Real-time PCR System (Applied Biosytems). The level of gene expression was obtained using the ΔΔCt (Ct: threshold cycle) method in which all samples are first normalized to the level of 18S rRNA in each sample. Student's *t*-test or One-way ANOVA was used for statistical analysis of RT-qPCR results and *P* value less than 0.05 was considered significant. Primer sequences were indicated in Supplementary Table 1.

### Western blotting and Immunofluorescence staining

Total proteins were extracted from cells using RIPA buffer (Millipore) plus 0.1% SDS. Protein concentration was measured by BCA Protein Assay Kit (Thermo Scientific). HRP-conjugated secondary antibodies were purchased from DAKO. Signals were visualized with Super Signal West Pico Chemiluminescent Substrate Kit (PIERCE). For myotube immunofluorescence staining, differentiated cells were fixed with 4% paraformaldehyde, washed, and permeabilized with 0.5% Triton X-100. Then, cells were blocked with 10% goat serum, followed by incubation with MHC Ab (MF20; 1:200), secondary Ab Alexa Fluor 488 donkey anti-mouse IgG and nucleus were counter stained with DAPI. The following primary antibodies were used: COUP-TFII, pan-MHC (MF20), Npnt (R&D); eMYH3, myogenin, p-FAK, FAK, Caveolin 3 (Santa Cruz Biotech.); neoMYH8 (Novus); Integrin β1D (Abcam); GAPDH (Cell Signaling).

### Chromatin-immunoprecipitation (ChIP) analysis

ChIP was performed in regular cultured C2C12 cells following the protocol provided by EMD Millipore using a specific anti-COUP-TFII antibody (R&D) and control anti- mouse IgG (Sigma) as described previously^41^. Bound fraction and input DNA were subjected to SYBR Green based qPCR using the following primers: Npnt (forward) 5′-ATTTCTTGTCAATGGCTCGG-3′, (reverse) 5′-TCTACCGCACAGGAAAGTCA-3′; *Itgb1* (forward) 5′-TTGTGATTTGGTGATTCCGA-3′; (reverse) 5′-TCCCGGAATTAAAGCTTCCT-3′; *Cav3* (forward) 5′-AAGCCAGTCTCCTCTCCTCC-3′, (reverse) 5′-AAGCCAGTCTCCTCTCC TCC-3′; ChIP control primer (forward) 5′-TCCAGTCTCTCCCTGTGCTT-3′, (reverse) 5′-GCCTCCAAGAGGAAGGAACT-3′. The enrichment of peaks was represented as percentage of input using standard curve method. Standard curves were generated by serial dilution of input. All the results are obtained from 3 repeats and statistical significance was determined by Student’s *t* test. *P* value less than 0.05 was considered significant.

### Statistical Analysis

All experiments were performed using at least 4 paired mice or 3 independent repeated experiments from cells. Data are presented as mean ± SEM. Statistical significance was calculated using a 2-tailed Student’s *t* test for two sample comparison. One-way ANOVA followed by post-hoc tests (Bonferroni's Multiple Comparison Test) for experiments with more than 2 groups. Statistical significance is signified in the figure legends as *P < 0.05, **P < 0.01, and ***P < 0.001.

## Electronic supplementary material

Supplementary Figures
