# Supplementary material for: Dysregulation of nuclear receptor COUP-TFII impairs skeletal muscle development
Source: Sci Rep. 2017 Jun 9;7:3136. doi: 10.1038/s41598-017-03475-5 (PMC5466650; doi:10.1038/s41598-017-03475-5)
Supplement: Supplementary file 1 — Supplementary Figures [file 41598_2017_3475_MOESM1_ESM.pdf]

Dysregulation of nuclear receptor COUP-TFII impairs skeletal muscle development

Hui-Ju Lee[1], Chung-Yang Kao[1], Shih-Chieh Lin[1,4], Mafei Xu[1], Xin Xie[1], Sophia Y. Tsai[1,2,3,]*and Ming-Jer Tsai[1,2,3,]*

[1]Department of Molecular and Cellular Biology, [2]Program in Developmental Biology, [3]Department of Medicine, Baylor College of Medicine, Houston, TX 77030, U.S.A. [4]Department of Physiology, College of Medicine, National Cheng Kung University, Tainan 701, Taiwan R.O.C.

[*] Correspondence:
Sophia Y. Tsai, Ph.D. and Ming-Jer Tsai, Ph.D.
Department of Molecular and Cellular Biology
Baylor College of Medicine
One Baylor Plaza, Houston Texas, 77030
stsai@bcm.tmc.edu (SYT) or mtsai@bcm.tmc.edu (MJT)
Phone: 713-798-6253 (MJT); 713-798-6251 (SYT)
Fax: 713-798-8227

Running Title:
COUP-TFII represses Nephronectin expression and FAK activity.

Supplementary Table 1. Primer sequences for RT-qPCR

RT-qPCR	Primer Sequences
**mCOUP-TFII**	5’-ATGGACCACATACGGATCTTCC-3’; 5’-ACAGGCATCTGAGGTGAACAGG-3’
**mPax7**	5’-CTCAGTGAGTTCGATTAGCCG-3’; 5’-AGACGGTTCCCTTTGTCGC-3’
**mMyf5**	5’-CACCACCAACCCTAACCAGAG-3’; 5’-AGGCTGTAATAGTTCTCCACCTG-3’
**mMyomaker**	5’-TTCCTCCCGACAGTGAGCAT-3’; 5’-GCACAGCACAGACAAACCAG-3’
**mMyoD1**	5’-CGGGACATAGACTTGACAGGC-3’; 5’-TCGAAACACGGGTCATCATAGA-3’
**mMyogenin**	5’-CGATCTCCGCTACAGAGGC-3’; 5’-GTTGGGACCGAACTCCAGT-3’
**mMRF4**	5’-ATTCTTGAGGGTGCGGATTTC-3’; 5’-CCTTAGCAGTTATCACGAGGC-3’
**mMYH2**	5’-GACCTATCTG CTAGAGAAGTCC-3’; 5’-CAGCAGCATTTCGATCAGCTC-3’
**mMYH3**	5’-TCACCATGCTGTGAACGCTCTC-3’; 5’-AGTGCTGTCTTGGTAGCTTTGTA-3’
**mMYH8**	5’-CAACAAGTGTATAATGCGGTGG-3’; 5’-GCTGCTTGGTGTCCAGCTGC-3’
**mCkm**	5’-AGACAAGCATAAGACCGACCT-3’; 5’-AGGCAGAGTGTAACCCTTGAT-3’
**mNpnt**	5’-TGTCTCAACGGATACATGCTGC-3’; 5’-TCACAGCCATACTGACAGTTTG-3’
**mItgβ1D**	5’-TTCATGACAG AAGGGAATTT GC-3’; 5’-ACCAGCTTTACGTCCATAGTTTG-3’
**mCav 3**	5’-TTGAAGACGTGATTGCGGAG-3’; 5’-ACCCAGCAGTGTAGACAACAG-3’
**m18S rRNA**	5’-GTAACCCGTTGAACCCCATT-3’; 5’-CCATCCAATCGGTAGTAGCG-3’

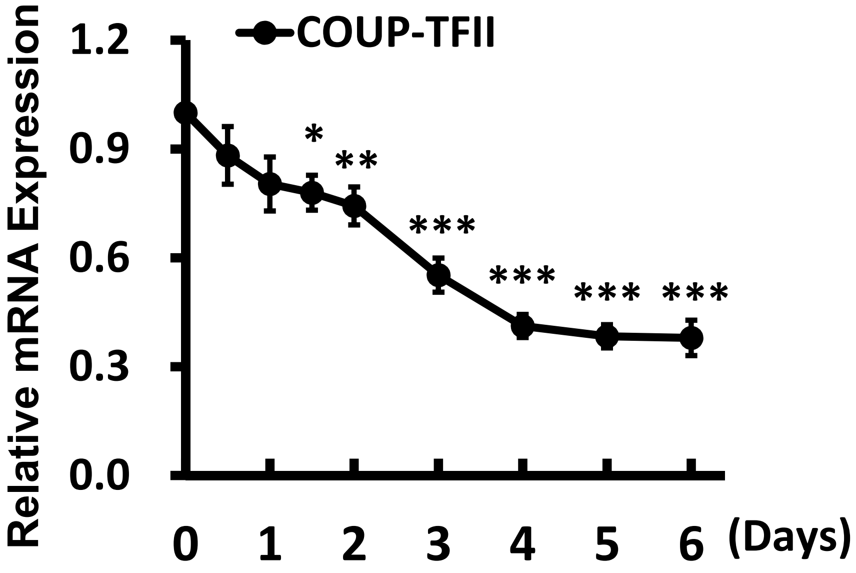

Supplementary Figure S1. Quantitative RT-PCR examination of COUP-TFII mRNA expression during myogenic differentiation in C2C12 cells. C2C12 cells were placed in the differentiation medium (DM, 2% horse serum) and incubated for the indicated time points. Data are shown as mean ± SEM. One-way ANOVA followed by Bonferroni's Multiple Comparison Test; * $p<0.05$, ** $p<0.01$, *** $p<0.001$, compared to day 0.

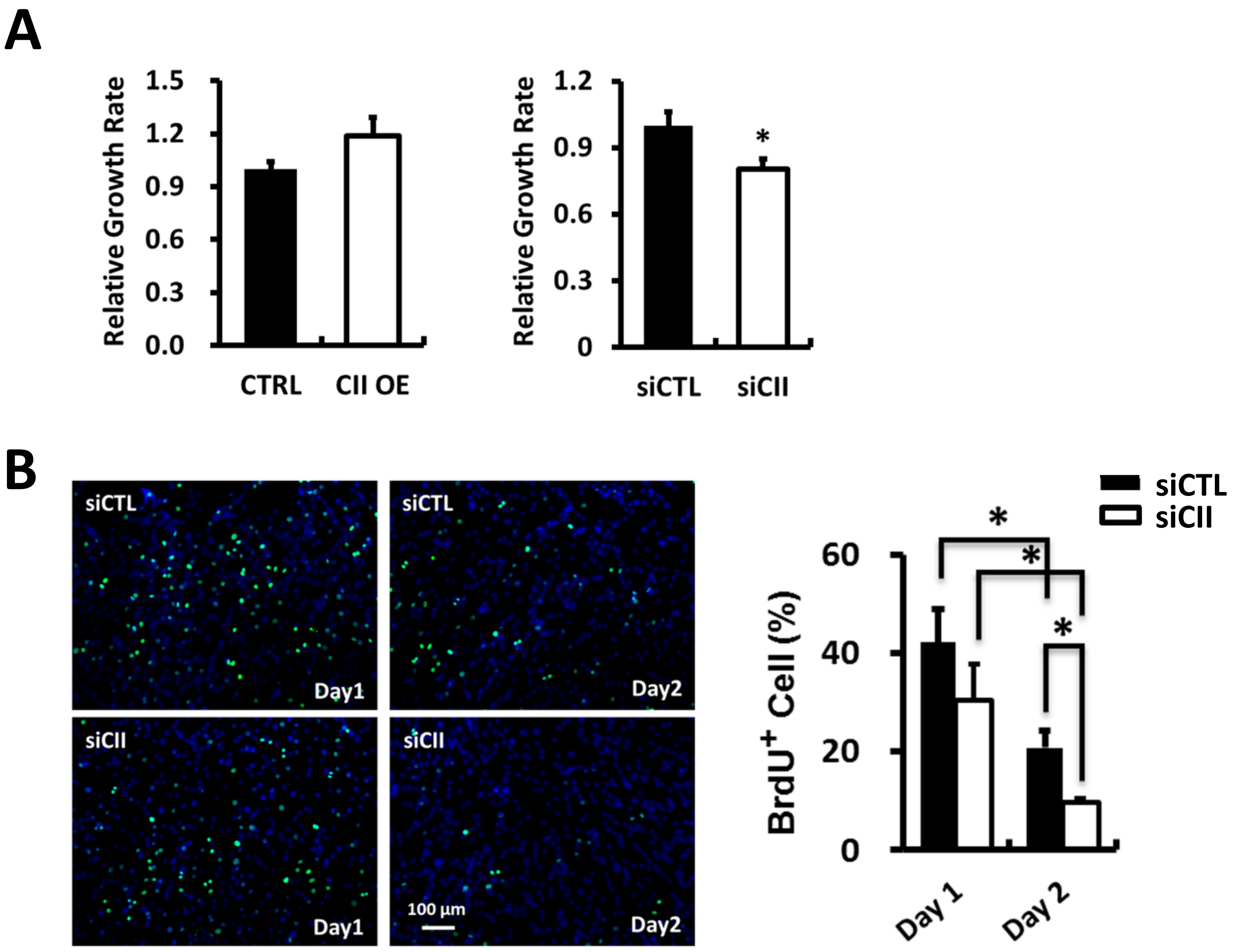

Supplementary Figure S2. (A) MTS examination of the growth rate of COUP-TFII overexpressing and COUP-TFII silencing myoblasts. Same amount of cells were seeded in 10% FBS culture medium for 2 days and the relative cell number were measured by O.D.490. **(B)** BrdU incorporation assay measured the proliferation rate of COUP-TFII silencing cells during differentiation. 10 µM BrdU was added at indicated post-differentiation time points and incubation for 12 hours followed by anti-BrdU immunofluorescence staining. Right panel shows the quantified results. Data are shown as mean ± SEM. Two-tailed Student's t-test; * p<0.05.

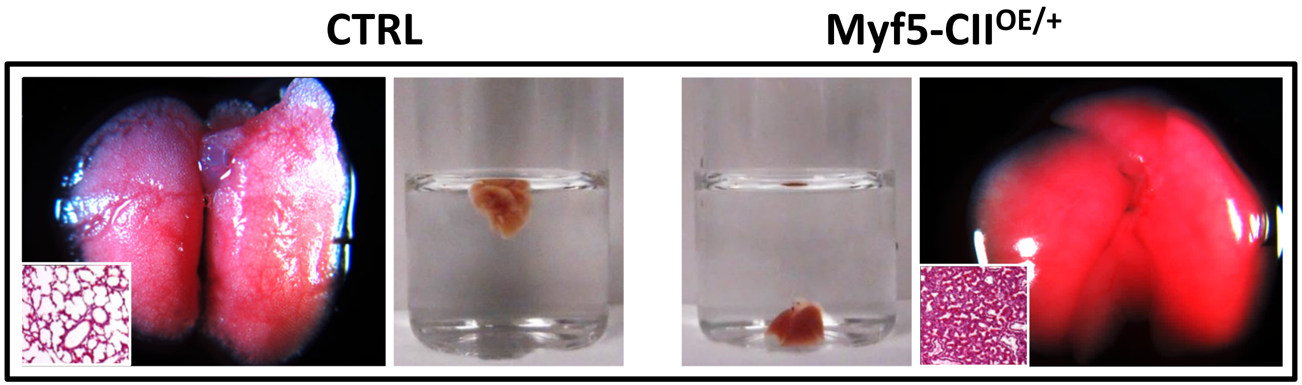

Supplementary Figure S3. Control lungs float, *Myf5-CII*$^{OE/+}$ lungs sink to the bottom which means *Myf5-CII*$^{OE/+}$ lungs were not inflated with air. Lung tissues H&E staining were shown in the bottom square.

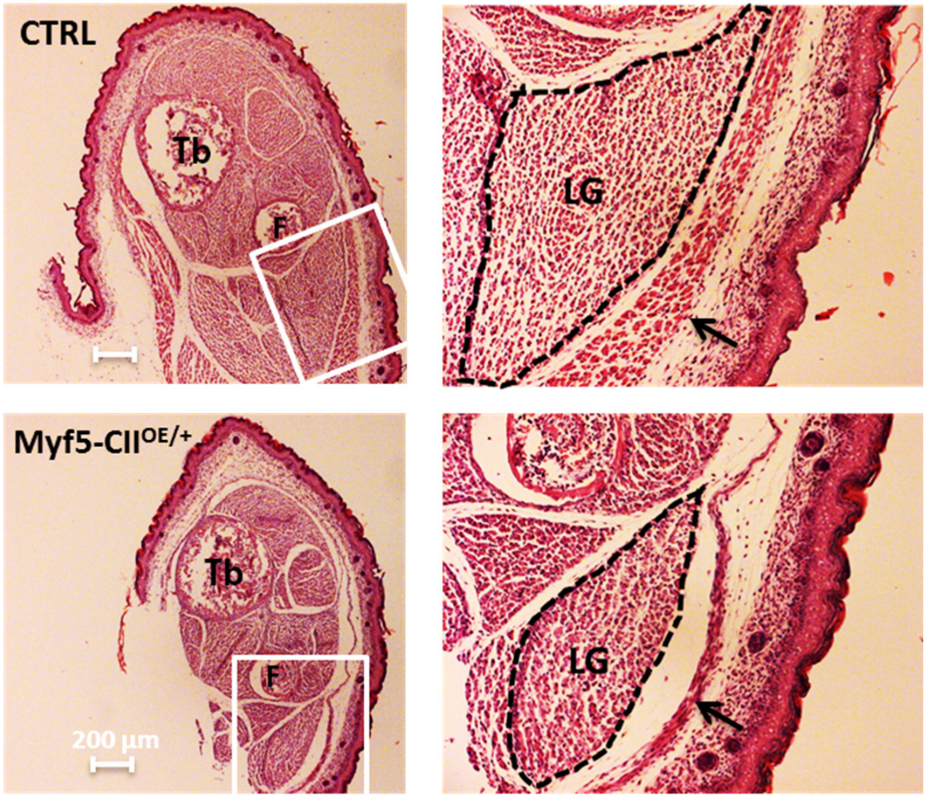

Supplementary Figure S4. H&E staining of hind-limb cross-section of E18.5 embryo. Enlarged square area of the gastrocnemius lateralis muscle was shown in the right panel. **Tb:** Tibia, **F:** Fibula, **LG:** gastrocnemius lateralis muscle. **Arrow:** biceps femoris muscle.

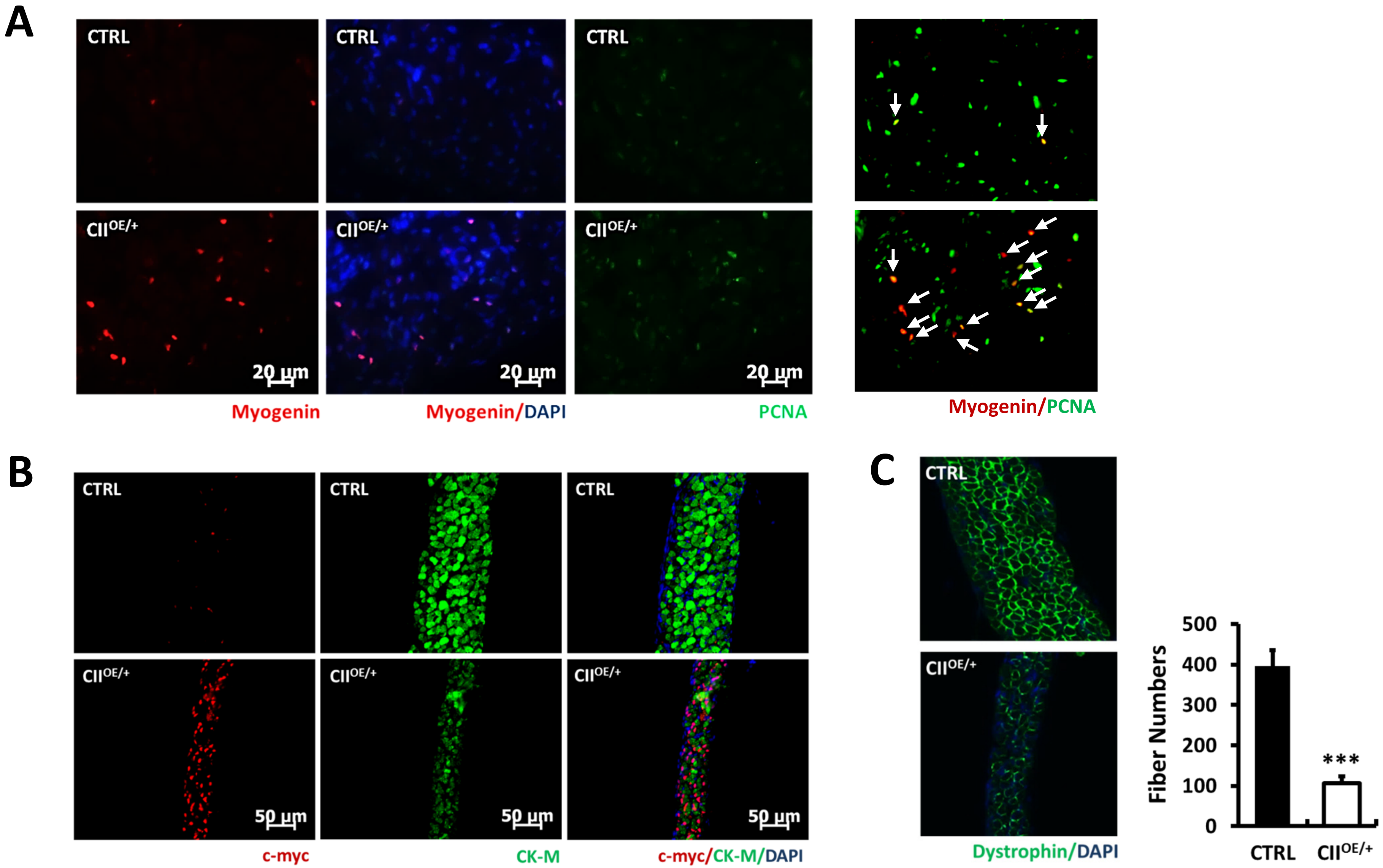

Supplementary Figure S5. Immunofluorescence staining showing that **(A)** Proliferating Myogenin^{+} myoblasts were increased; **(B)** muscle form Creatinine Kinase (CK-M) and **(C)** muscle fibers were reduced in the diaphragm muscle of *Myf5-CII$^{OE/+}$* mice. Right panel shows the quantitative fiber number/field. N=4. PCNA: proliferating cell nuclear antigen. Arrow head: Myogenin^{+}/PCNA^{+} cells. C-myc stained the ectopic overexpressed COUP-TFII. Dystrophin stained the periphery of muscle fibers.

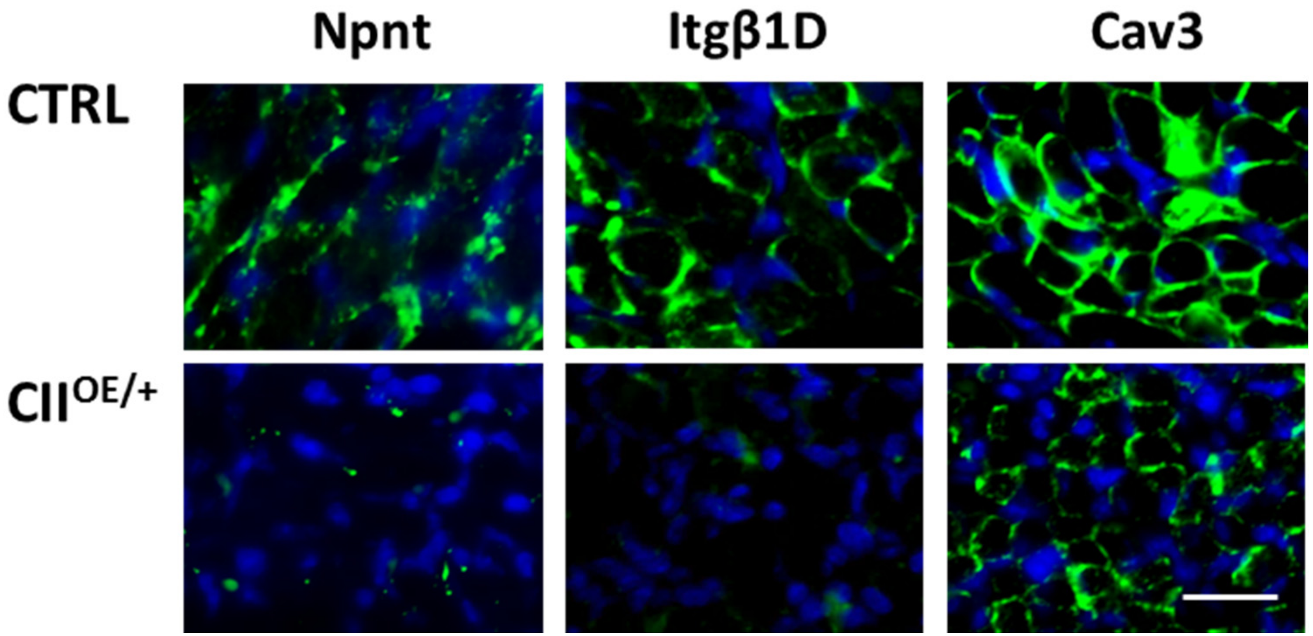

Supplementary Figure S6. Immunofluorescence staining of Nephronectin, Integrin β1D and Caveolin 3 protein levels in the skeletal muscle of E18.5 control and *Myf5-CII*$^{OE/+}$ embryo. Green fluorescence stained for the indicated proteins and DAPI was used for nuclear counterstain. Scale bar= 20 µm.

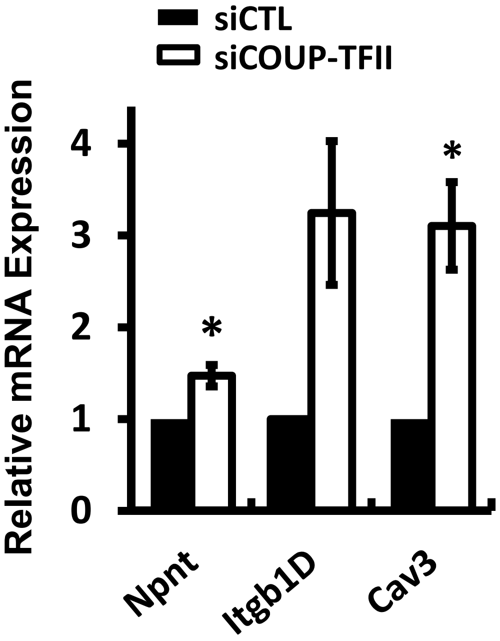

Supplementary Figure S7. Quantitative RT-PCR examination of Nephronectin (Npnt), Integrin β1D (Itgb1D) and Caveolin 3 (Cav3) mRNA expression in COUP-TFII silencing C2C12 cells before induction (Day 0). Data are shown as mean ± SEM. Two-tailed Student's *t*-test; * $p<0.05$, compared to control cells.

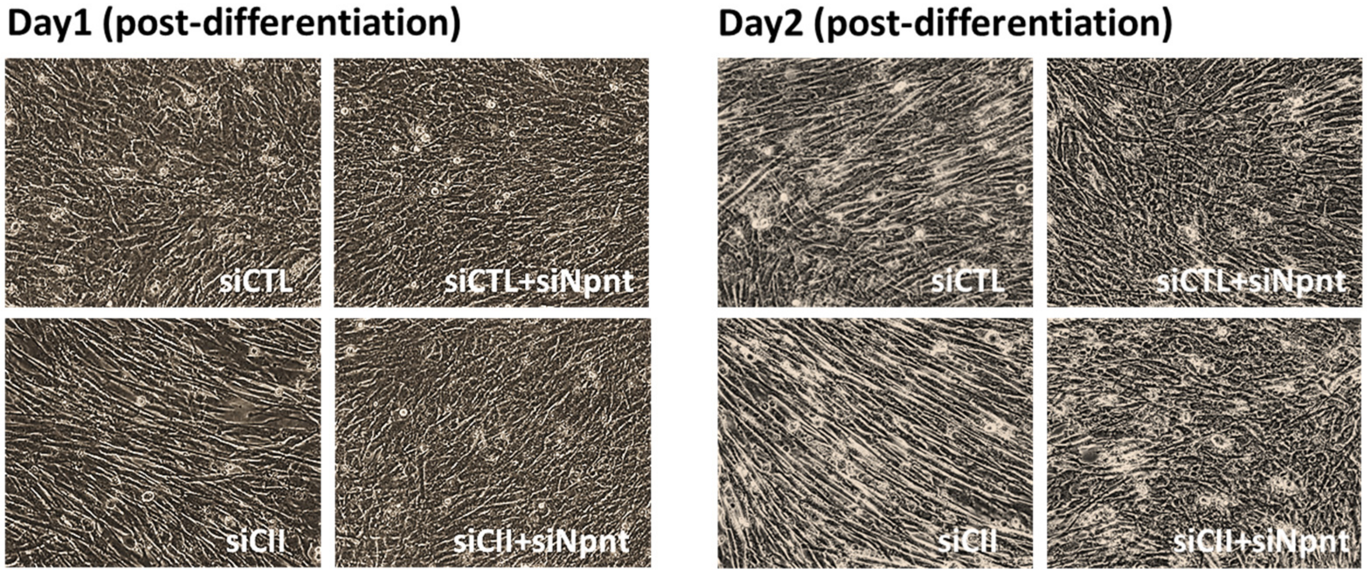

Supplementary Figure S8. Phase contrast images of control, COUP-TFII silencing, Npnt silencing and double silencing C2C12 cells one day and two days post-differentiation. Cells were pre-silencing of indicated genes 3 days and reseeded before serum withdrawal.
